# Commentary: Interpretations without justification: a general argument against Morgan's Canon

**DOI:** 10.3389/fpsyg.2016.00452

**Published:** 2016-03-30

**Authors:** Eduardo Mercado

**Affiliations:** Department of Psychology, University at Buffalo, The State University of New YorkBuffalo, NY, USA

**Keywords:** comparative cognition, anthropomorphism, anthropocentrism, animal psychology, philosophy of science, epistemology

Morgan's Canon has been touted as “the most awesome weapon in animal psychology,” (Wynne and Udell, 2013, p. 14). The enemies that this century-old principle is usually employed to destroy are explanations of behavior that potentially exaggerate the cognitive capacities of nonhumans. Often, the battle is between explanations based on associative learning and explanations that invoke other “more sophisticated” psychological processes (Shettleworth, 2010; Heyes, 2012; Smith et al., 2012), where more sophisticated typically means evident in adult humans. Given the longevity and apparently foundational importance of Morgan's Canon, some comparative psychologists might be surprised to learn that philosophers have recently argued that this principle is illegitimate as a basis for choosing between competing explanations of animal behavior (Fitzpatrick, 2008; Heyes, 2012; Buckner, 2013; Starzak, 2016). Starzak (2016), in particular, suggests that Morgan's Canon should be jettisoned in favor of more general scientific principles shared by all disciplines. This commentary considers the merit of Starzak's argument against Morgan's Canon.

Morgan's (1903, p. 59) Canon states, “In no case is an animal activity to be interpreted in terms of higher psychological processes, if it can be fairly interpreted in terms of processes which stand lower in the scale of psychological evolution and development.” Historically, this statement has been interpreted in disparate ways (Sober, 1998, 2005; Thomas, 1998, 2001; Allen-Hermanson, 2005; Smith et al., 2012; Shettleworth, 2013). For instance, Morgan's “lower” and “higher psychological processes” have been portrayed as corresponding to simpler vs. more complex cognitive abilities, implicit vs. explicit memory capacities, reflexive vs. volitional processes, etc. Similarly, because the phylogenetic scale that Morgan envisioned is archaic, modern interpretations of Morgan's Canon often adopt alternative evolutionary continua. Shettleworth (2010, 2013), for example, describes cognitive capacities demonstrated in many species (and therefore presumed to be phylogenetically older) as “lower processes,” reserving the higher end of the scale for evolutionarily younger, species-specific specializations (see also Sober, 1998, 2005; Karin-D'arcy, 2005).

The crux of Starzak's (2016) argument against Morgan's Canon is that regardless of which interpretation of the principle one favors, there is no justification for assuming that explanations emphasizing processes at one position along *any* particular “scale of psychological evolution and development” are better than other explanations that emphasize processes at other positions along that scale. By analogy, systematic rankings of human beauty vary across different cultures and generations such that an individual may be ranked high on one scale and low on another. But, regardless of which criteria for ranking beauty one chooses, there are no rational or empirical grounds for claiming that all “ugly” individuals are inherently better than those judged to be beautiful.

Some have argued, however, that there are justifications for preferring explanations that occupy the lower end of psychological scales. For example, Karin-D'arcy (2005) defends a modernized version of Morgan's Canon by claiming that the principle forces one to dig deeper, encourages careful interpretation, protects from error and misjudgment, provides a check against anthropomorphism, emphasizes the importance of cross-species differences, prevents reliance on hastily drawn conclusions, and promotes closer assessment of test validity. Evidence for such claims is currently lacking and philosophers who have considered these potential justifications of Morgan's Canon have found them to be weak or invalid (Fitzpatrick, 2008; Starzak, 2016). Possible upgrades to Morgan's Canon have been proposed (Andrews and Huss, 2014; Meketa, 2014), but Starzak (2016) advocates only giving preference to explanations of animals' actions that have greater evidential support (see also Sober, 2005; Fitzpatrick, 2008; Heyes, 2012; Mikhalevich, 2015).

Unfortunately, evidence about the cognitive origins of animals' actions rarely justifies choosing one explanation over another. Naturalistic observations can reveal a subset of animals' actions that are salient to human observers, but rarely compel inferences about any mental processes that may accompany or enable those actions. Experimental data can reveal how some animals fare when faced with cleverly contrived scenarios, but are usually consistent with multiple, comparably evidenced, interpretations regarding what subjects represent, remember, or understand. The so-far unobservable qualities of mental processes are exactly what make scientists skeptical of claims about how animals cognize, and what historically have sparked intellectual battles between mechanists and cognitivists. Morgan's Canon leads to unjustified verdicts about animals' minds, but empiricism gives rise to deadlocked juries.

For Starzak (2016), preference for an explanation of an animal's actions is justified when that explanation can be shown to be better (e.g., greater in explanatory power or more likely to be true). In contrast, Morgan justified his Canon by claiming that, “The only fruitful method of procedure is the interpretation of facts observed with due care in the light of sound psychological principles,” (Morgan, 1903, p. 59). From Morgan's perspective, using sound principles to identify superior explanations enables scientific progress. Starzak (2016) and other critics of Morgan's Canon appear to implicitly endorse this position by focusing more on the soundness of candidate principles rather than challenging the claim that such principles are necessary. Although it might seem self-evident that interpreting facts using sound principles is an essential component of scientific research, historical analyses suggest that science advances despite, and in some cases even because of, reliance on scientifically unsound principles (Koestler, 1959; Feyerabend, 1975). For example, the anthropomorphic, anecdotal approach adopted by Darwin and Romanes (considered “unsound” by modern standards) helped to instigate the experimental studies conducted by Morgan and Thorndike, thereby sparking a revolution in psychological studies of animals. Objectively, it is difficult to tell whether Morgan's Canon has catalyzed or cannibalized progress in animal cognition research.

Despite its critics, Morgan's Canon seems likely to maintain its exalted status within the field of comparative cognition. Textbooks may continue noting that there are “some concerns” about the principle while simultaneously emphasizing its fundamental importance. Reviewers will probably persist in attacking interpretations that “break the rule.” Luckily for comparative psychologists, history shows that adherence to general methodological or theoretical principles can lead to advances in understanding even when those principles are specious. Nevertheless, it remains possible that slavish adherence to this particular unsound principle could impede future progress by systematically biasing researchers toward underattributing cognitive capacities to nonhumans (Fitzpatrick, 2008; Mikhalevich, 2015).

## Author contributions

The author confirms being the sole contributor of this work and approved it for publication.

### Conflict of interest statement

The author declares that the research was conducted in the absence of any commercial or financial relationships that could be construed as a potential conflict of interest.
